# Motor Speed Matters! Cognitive Profile of Parkinson’s Disease Patients With and Without Deficits in Motor Speed

**DOI:** 10.4103/0028-3886.317232

**Published:** 2021-05-01

**Authors:** Vidya Menon, Shantala Hegde, PV Pratyusha, Nitish Kamble, Ravi Yadav, Amitabh Bhattacharya, Pramod K Pal

**Affiliations:** 1 Department of Neurology, National Institute of Mental Health and Neuro Sciences; 2 Clinical Neuropsychology and Cognitive Neuro Science Center, Department of Clinical Psychology, National Institute of Mental Health and Neuro Sciences; 3 Department of Biostatistics, National Institute of Mental Health and Neuro Sciences, Bengaluru, Karnataka, India

**Keywords:** Cognitive deficits, finger tapping test, motor speed, neurocognitive profile, Parkinson’s disease

## Abstract

**Background:**

Parkinson’s disease (PD) is characterized by bradykinesia, tremor, rigidity, postural instability and cognitive deficits in attention, executive functions, learning and memory. Motor speed, measured using Finger Tapping Test (FTT), is an important indicator and predictor of cognitive and motor functions. Deficits in motor speed have significant impact on performance on other neuropsychological tests.

**Objective:**

This study aimed to understand and compare the cognitive profile of patients with and without deficits in motor speed as evaluated on the FTT.

**Method and Material:**

A detailed neuropsychological evaluation using the NIMHANS Neuropsychological Battery was carried out on 70 PD patients. The PD patients were divided into patients with (*n*= 46) and without (*n*= 24) motor speed deficits. The two groups were comparable with regard to age (*P*= 0.591), years of formal education (up to 10^th^– 24.3, above 10^th^– 75.7) duration of illness (*P*= 0.703) and age of onset (*P*= 0.721).

**Results:**

Across the various cognitive domains such as executive functions, verbal recognition, visuospatial functions, visual learning and memory, the group without deficits in motor speed performed significantly better in comparison to patients with motor symptoms.

**Conclusion:**

A short and simple test such as FTT may be helpful in predicting the range and severity of cognitive deficits across other cognitive domains in patients with PD. Future studies on larger cohort examining the intricate role and association of FTT and other motor functions such as dexterity may be helpful in understanding the nature and severity of other cognitive functions in this clinical population.

Motor speed is a function that involves motor and cognitive domains of brain.^[1]^Finger Tapping Test (FTT), is a universally used neuropsychological tool, commonly employed to quantitatively assess motor performance in upper extremities. Studies have indicated that motor speed deficits can be considered as a marker for evaluating motor and cognitive functioning and that both are related.^[2]^Present study compares the cognitive profile of PD patients with (WD) and without deficits (WOD) in motor speed as evaluated on FTT and thereby understand the predictive value of motor speed in cognitive functioning.

## Methods

The present study included 70 PD patients. Patients were recruited from the inpatient, outpatient and Movement disorders clinic of the department of Neurology, National Institute of Mental Health and Neurosciences (NIMHANS), a tertiary care hospital in Bengaluru, India. The study period was from January 2018 to June 2019. Ethical approval was obtained from the institute ethics committee. All the patients gave written informed consent. The recruited patients were referred for detailed neuropsychological evaluation as part of a larger ongoing funded research project or as part of routine clinical care.

Patients less than 75 years of age diagnosed as PD as per the United Kingdom Parkinson’s disease society brain bank diagnostic criteria,^[3]^with formal education more than 7^th^std were included in the present study. The patients with other coexisting neurological illness were excluded from the study.

All the patients were evaluated with detailed clinical history and examination. Unified Parkinson’s disease rating scale, part III (UPDRS-III)^[4]^was used to evaluate motor disability while handedness was assessed using Edinburgh handedness inventory.^[4,5]^The research design used in the study was cross sectional. A detailed neuropsychological evaluation using selected tests from NIMHANS Neuropsychological battery^[6]^and Wechsler memory scale III (WMS)^[7]^was carried out on 70 PD patients. The scores obtained on the neuropsychological tests were compared with Indian norms appropriate to patients’ gender, age and education.^[6]^A score below the 15^th^percentile score (1SD below the mean) is the cut off score.^[8]^The details of the test battery are mentioned in Table 1.

The obtained data was scored, coded and analyzed using Statistical Package for Social Sciences (SPSS) windows version 16.^[9]^The variables were tested for normality using Shapiro-Wilk test. Variables that fell in the normal range in both groups or in at least one group (WOD) were compared using the parametric test (independent sample t test). Whereas, the other variables which were not following normality in both the groups were compared using non parametric test (Mann–Whitney test). The Mann–Whitney test compares the number of times a score from one sample is ranked higher than a score from another sample and it detects the statistical significance by comparing the median and inter quartile range in the two groups.

## Results

The present study included 70 PD patients. The clinical details are provided in Table 2. The sample was divided into two categories: with motor speed deficits (WD) and without motor speed deficits (WOD) based on their performance scores in FTT (*n*= 46 WD and *n*= 24 WOD). Results indicated that in most of the cognitive domains such as attention, executive functions (fluency, planning and problem solving, set shifting), visuospatial functions, visual learning and memory there was a significant difference among the two groups. The results are provided in Table 3.

Further, based on the bivariate analysis results, an attempt was also made to find the predictors of cognitive domains, and the domains that correlated with both right and left finger tapping scores were considered for the regression analysis. Multiple linear regressions were performed to identify whether finger tapping (right and left) score will predict the cognitive domains and results indicate that finger tapping scores can predict attention and executive functions, however as the R^2^is less, results should be interpreted with caution. Table 3delineates the results of predictive analysis. Please refer to Table 4.

## Discussion and Conclusion

In the current study, on comparing the cognitive profile of WD and WOD patients, results indicated that in most of the cognitive domains such as verbal and category fluency, planning and problem solving, set shifting, verbal recognition, visuospatial functions, visual learning and memory there was a significant difference among the two groups. PD is a neurological disorder associated with dopamine depletion in the basal ganglia and this dopamine loss results in frontal disconnections and this is directly related to the manifestation of motor and cognitive symptoms in PD.^[10]^A study included comprehensive neuropsychological assessment such as tests of executive functions, memory, psychomotor speed, attention, visuospatial, and language functions to compare 94 PD patients with 84 healthy controls, and it concluded that PD patients performed significantly worse on executive functions (i.e., category of card sorting) and psychomotor speed (i.e., processing speed index).^[11]^A study that was undertaken on PD patients from India, reported deficits in executive functions on screening tool such as the Frontal Assessment Battery (FAB) developed by Alexander Luria.^[12]^This study included 170 patients with PD and observed that levels of formal education correlated significantly with the observations on the FAB. A study on relationship between motor impairment and cognitive impairments in PD had also revealed that the severity of motor impairment positively correlated to impairment on cognitive domains such as memory, language, visuospatial, and frontal lobe functions.^[13]^

Deficits in motor speed is found to have major impact on the performance on other neuropsychological tests and clinicians often keep this in mind before interpreting the performance of patients on other neuropsychological tests. A study on 170 elderly patients (83 men, 87 women; *M*age = 82.1 yr., *SD*= 6.2) underwent cognitive assessment and 15 seconds of finger tapping and results indicated a significant increase in the length and variability of the finger- touch phase among participants with mild cognitive impairment or dementia compared to participants who did not have cognitive impairment, thus suggesting a relationship between finger tapping and attention, short-term memory and cognitive functions.^[14]^In Alzheimer’s disease (AD) and mild cognitive impairment (MCI) patients it was found that, decreased finger dexterity was associated with decline in cognitive function and thus it can be a marker for cognitive functions.^[15]^Motor speed has also been shown to predict the specific and general deficits of verbal fluency, set shifting, reasoning, executive functions, and attention of both bipolar-I and patients with schizophrenia because of a common pathogenic factor related to psychomotor slowness. Thus, motor speed appears to be an appropriate endophenocognitype for schizophrenia and bipolar disorder.^[16]^

FTT has been used to measure outcome in stroke patients, who did not present any clinical motor deficits of the preferred hand. Stroke-related action slowing is mainly due to slowing of perceptual and motor processes. Action slowing was related to lesions of the large network. In FTT the lesion location was in the left middle frontal gyrus and lenticulate nucleus. Further, FTT performance predicted outcome, over and above what other motor and perceptual tasks contributed, suggesting the action slowing, and thus FTT was considered as a promising prognosis index.^[17]^

A study found that patients with MCI, AD, and PD all have abnormalities in finger tapping as compared to healthy adult controls. During a repeated ten-second response window, patients with AD and MCI produced the fewest number of finger taps, while patients with PD produced even more than cognitively healthy older adults. In addition, AD and MCI individuals had the longest inter-tap interval, while the PD patients had the shortest, and this was similar to cognitively healthy older adults.^[18]^

In the current study, domains such as mental speed, focused attention and amount of time taken in planning task, the group WOD in motor speed has performed significantly better than the group WD motor speed deficits. However, as there could be a direct influence of the motor speed deficit in these three domains, which was evaluated using timed tests, the results are not highlighted. On the other hand, from the performance on the non-motor tasks measuring executive functions, such as in COWA, ANT and in untimed tasks such as the total number of problems solved in TOL test, WCST and the visual construction and visual learning tasks such as in CFT it can be seen that the difference in the significance of deficits between the two groups is prominent. A significant correlation between cognitive domains and motor slowing was observed in a similar study and the slowness in PD was seen in motor domain, cognitive mental operations and in domains of behavior.^[19]^

The predictive analysis result in this study has indicated that the right finger tapping score predicted CT2 and COWA scores, and a slight inclination towards prediction was also observed in right finger tapping score obtained in DS and DSS, and in left finger tapping score in ANT. Thus, the finger tapping score was found to predict attention and executive functions. Motor speed has been shown to predict the specific and general deficits of verbal fluency, set shifting, reasoning, executive functions, and attention of patients with bipolar-I and schizophrenia^[16]^and has been recognized as an important indicator and predictor of cognitive and physical symptoms in PD.^[20]^Balancing skill and functional mobility in patients with PD were also found to be significantly correlated with executive functions, cognitive impairment and patient’s ability to switch attention between two tasks.^[21]^However, in contrast to most of these studies, a study on predictors of cognitive impairment in advanced PD, found that older age and tremor at the onset were significant predictors of poor cognitive performance. Tremor was explained as a marker for more widespread brain pathology that contributes to an increased risk of cognitive impairment, than predominant akinesia/rigidity.^[22]^

Motor speed is found to be an important determinant for cognitive functions and deficits in motor and cognitive domains have been found to be closely interrelated. Thus, a short and simple test such as FTT may be helpful in predicting the range and severity of cognitive deficits across other cognitive domains in patients with PD. Future studies on larger cohort examining the intricate role and association of finger tapping speed and other motor functions such as reaction time, dexterity may be helpful in understanding the nature and severity of cognitive functions in this clinical population.

## Figures and Tables

**Table 1:** Neuropsychological tests, their respective domains and functions

Domain	Cognitive functions	Test
Speed	Motor speed	Finger Tapping Test (FTT)
	Mental speed	Digit Symbol Substitution Test (DSS)
Attention	Focused Attention	Color Trail (CT)
	Sustained Attention	Digit Vigilance Test (DVT)
Executive Functions	Verbal Fluency	Controlled Oral Word Association Test (COWA)
	Category Fluency	Animal Names Test (ANT)
	Planning	Tower of London (TOL)
	Concept formation and Set Shifting	Wisconsin Card Sorting Test (WCST)
	Response Inhibition	Stroop Test (ST)
	Working memory	Verbal N back Test
		Digit Span Test (DS)
		Spatial Span Test (SS)
Learning and Memory	Verbal	Auditory Verbal Learning Test (AVLT)
	Visual	Complex Figure Test (CFT)
Visuo spatial Construction	Copy trial of the test	Complex Figure Test (CFT)

**Table 2:** Demographic profile and clinical characteristics of the patients

Cognitive Domain	Mean (SD)		***t***/*U*	***P***
	WOD (*n*=24)	WD (*n*=46)			
Age (years) Age at onset (years)	56.33 (9.07) 50 (10.73)	57.63 (9.77) 51 (10.75)	-0.54 -0.359	0.591 0.721
	**Median (Q1, Q3)**			
Duration (years)	7 (1.88, 10)	5 (3, 10)	477	0.703

WOD: Without motor deficits, WD: With motor deficits

**Table 3:** Comparison of Cognitive domains among WOD and WD groups

Cognitive Domains	Mean (SD)		***t**/*U**	***P***
	WOD (*n*=24)	WD (*n*=46)		
LT score	43.01 (6.98)	29.9 (6.6)	7.736	<0.001
CT2	192.54 (63.81)	257.8 (103.62)	-3.251	0.002
COWA	8.92 (3.56)	6.65 (3)	2.814	0.006
ANT	12.67 (3.28)	9.87 (2.86)	3.695	<0.001
CFT DR	16.5 (6.3)	12.96 (7.23) (*n*=45)	2.026	0.047
TNMM	8.92 (1.93)	7.7 (1.94)	2.501	0.015
	**Median (Q1, Q3)**			
DSS	294.5 (225.75,336.75)	396 (301,479.25) (*n*=42)	280.5	0.003
2MT	5 (4,6)	6.25 (4.5,8.5)	374.5	0.027
3MT	10.25 (8,19.5)	16.75 (11.59,24.56)	322	0.004
5MT	26.88 (18,35.75)	36.53 (27.31,65.38)	332.5	0.007
5NMM	2 (1,3)	1 (1,1)	300.5	0.001
% PR	15.24 (10.58,23.3)	20.75 (14.1,32.98) (*n*=42)	346.5	0.036
PE	13 (9,25)	24.5 (14.75,38.5) (*n*=42)	347	0.036
% PE	14.15 (10.58,19.5)	20.3 (14.01,30) (*n*=42)	347	0.036
% CLR	66.45 (43.35,74.08)	46.05 (22.03,68.15) (*n*=42)	344.5	0.033
False Alarm	0 (0,0)	0 (0,1)	412.5	0.037
Copy	34.5 (33,36)	32 (25.5,34.75) (*n*=45)	324	0.006
CFT IR	16.25 (15.13,24.25)	13.5 (6.75,20) (*n*=42)	367	0.029

WOD: Without motor deficits, WD: With motor deficits, LT:Left Finger tapping, CT2: Color trail2, COWA: Controlled Oral Word Association Test, ANT: Animal Names Test, TNMM: Total number of minimum moves, DSS: Digit Symbol Substitution, MT: Mean time, NMM: Number of minimum moves, PR: Perseverative response, PE: Perseverative error, %CLR: Conceptual level response, CFT IR: Complex Figure Test Immediate recall

**Table 4:** Predictive analysis of finger tapping (Right and Left) scores

Dependent Variable (DV)	Predictor	β (SE)	***t***	***P***	***R*** ^2^
CT2	R	-5.068 (1.69)	-3.002	0.004	0.131
	Lt	1.828 (1.79)	1.024	0.309	
	Constant	355.36 (44.66)	7.957	<0.001	
COWA	Rt	0.124 (0.06)	2.054	0.044	0.079
	Lt	-0.017 (0.06)	-0.261	0.795	
	Constant	3.531 (1.597)	2.211	0.030	
ANT	Rt	0.065 (0.055)	1.185	0.240	0.189
	Lt	0.106 (0.058)	1.823	0.073	
	Constant	4.810 (1.460)	3.294	0.002	
Total	Rt	0.065 (0.25)	0.263	0.793	-0.002
	Lt	0.178 (0.260)	0.685	0.496	
	Constant	38.324 (6.499)	5.897	<0.001	
DS	Rt	0.120 (0.06)	1.887	0.064	0.090
	Lt	0.004 (0.07)	0.058	0.954	
	Constant	8.306 (1.71)	4.856	<0.001	
DSS	Rt	-6.846 (3.61)	-1.895	0.063	0.072
	Lt	0.576 (3.89)	0.148	0.883	
	Constant	623.987 (99.18)	6.292	<0.001	

CT2: Color trail2, COWA: Controlled Oral Word Association Test, ANT: Animal Names Test, DS: Digit span test, DSS: Digit Symbol Substitution Test

## Key Message

Neuropsychological assessment in Parkinson’s disease should be carried out with great caution owing pharmacological treatment (on-state and off-state) and tremors. A simple and less time-consuming test such as Finger Tapping test measuring motor speed could be a quick method to gauge the extent of cognitive deficits in patients with Parkinson’s disease.
